# Odynophagia after Cardiac Catheterization: A Rare Complication in the Presence of Aberrant Subclavian Artery

**DOI:** 10.1155/2020/7431726

**Published:** 2020-12-01

**Authors:** Moumita Naidu, Robby Singh, Marcel Zughaib

**Affiliations:** Providence Hospital-Michigan State University, USA

## Abstract

**Background:**

Vascular complications from transradial cardiac catheterization are uncommon. Mediastinal hematoma is a rare complication with life-threatening potential. We present a case of a patient who underwent cardiac catheterization and subsequently experienced odynophagia from injury to an aberrant subclavian artery that led to a mediastinal hematoma. *Case Report*. A 59-year-old female with past medical history of coronary artery disease presented with complaints of angina and underwent a transradial cardiac catheterization. Immediately after the procedure, the patient complained of chest pain and odynophagia. EKG and echocardiogram were unremarkable, and a CT scan of the chest demonstrated an ill-defined fluid collection present in the superior mediastinum and an aberrant right subclavian artery. The patient was closely monitored in the Intensive Care Unit, and the patient remained hemodynamically stable throughout the admission. The patient was subsequently discharged home in good condition and did well on outpatient follow-up.

**Conclusion:**

Vascular injuries associated with delivery of standard radial catheters in the subclavian artery are rare, with very few cases reported in the literature. We presented the case of a patient who had a previously unidentified right aberrant subclavian artery with a retroesophageal course which precipitated the hematoma and subsequently resulted in odynophagia despite an uncomplicated catheterization. This rare complication of a commonplace procedure necessitates prompt recognition, appropriate hemodynamic management, and possible repair of the injured vessel to appropriately manage a potentially life-threatening condition.

## 1. Introduction

Transradial cardiac catheterization is a commonplace procedure due to increased patient comfort and low rates of vascular complications. Radial artery occlusion is the most frequent complication (1.77%) though pseudoaneurysms (0.38%) and AV-fistulas (0.19%) can also be seen postprocedure [1, 4]. Potentially life-threatening vascular complications such as mediastinal hematoma are extremely rare and are usually due to trauma, laceration, or perforation of the thoracic arch vessels and can lead to significant morbidity [2, 3]. Previously unidentified anatomic variants, calcification, and rigid, large-bore catheters are some of the contributing factors which can predispose patients to vascular injuries.

Although patients with mediastinal hematoma typically present with chest pain, bleeding, or hemodynamic instability, this very rare albeit feared complication (.01% of cases) and carries a mortality rate is forty-nine percent within the first 24 hours [5]. Odynophagia is a rare presenting symptom for a patient with a mediastinal hematoma and should not be dismissed especially after a transradial catheterization. It is important for astute clinicians to familiarize themselves with not only the predisposing risk factors for vascular injuries but also the variety of symptoms which can lead to a diagnosis of mediastinal hematoma which is potentially fatal.

## 2. Case Report

A 59-year-old female with a past medical history of tobacco abuse, hypertension, and coronary artery disease and prior percutaneous coronary intervention presented with complaints of angina and subsequently underwent coronary angiography. The procedure was performed via the right radial artery approach. A 5 French Jacky catheter was used to engage the left coronary artery system, and a 6 French Judkins Right 4 catheter was used to engage the right coronary artery system. The procedure was completed without difficulty from the operator, and the patient was transferred to the postoperative holding area in stable condition.

**Figure 1:**
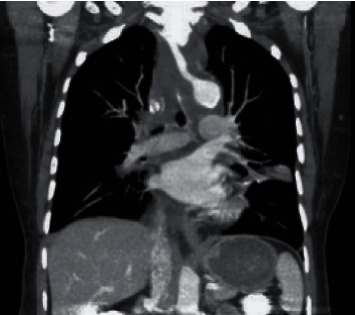
CT scan demonstrating ill-defined fluid collection in the superior mediastinum (arrow).

However, immediately after the procedure, the patient began to experience chest pain and odynophagia. Stat electrocardiogram and bedside echocardiogram were unremarkable, and a CT scan demonstrated an ill-defined fluid collection present in the superior mediastinum (Figure 1).

**Figure 2:**
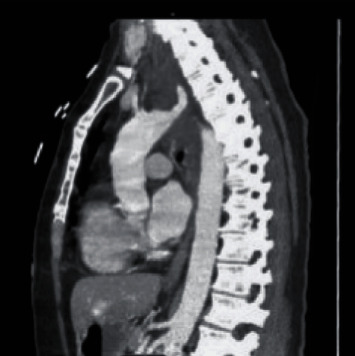
Aberrant right subclavian artery coursing posterior to the trachea and esophagus (arrow).

While a hematoma and a mass were both considered to be in the differential diagnosis, the CT scan also made note of an aberrant right subclavian artery (ARSA) coursing posterior to the trachea and esophagus (Figure 2). Esophageal anomalies were otherwise ruled out with an esophagram, and the differential diagnosis was narrowed down to a mediastinal hematoma.

Consequently, the patient was admitted to the cardiac care unit but continued to complain of midsternal chest pain and odynophagia. The patient remained hemodynamically stable and without any airway compromise. The patient was managed expectantly with analgesia. A repeat CT scan a day later again demonstrated the ill-defined collection which had now extended into the inferior right paravertebral aspect near the gastroesophageal junction. This was deemed to be an extension of the same fluid collection with the size of the hematoma remaining stable. The patient was monitored closely and improved clinically over the course of four days and was subsequently discharged home in good condition. On outpatient follow-up, the patient reported her symptoms had completely resolved.

## 3. Discussion

Vascular injury can be the result of a routine cardiac catheterization, but the injury associated with the delivery of standard radial catheters in the subclavian artery is rare, with only a few cases reported in the literature [1]. Most vascular complications occur due to laceration or perforation of thoracic arch vessels due to the tension created on the ostium of the artery from catheter manipulation. These previously reported cases highlight the importance of careful catheter placement and the use of fluoroscopy to confirm the relative position of the equipment during the procedure. In addition, choosing the right catheter type plays a vital role in preventing injury since rigid guide catheters, curved catheters, and larger French sizes are all associated with increased risk of vessel trauma. Calcified vessels are a separate risk which predisposes patients to vascular injuries, but most experienced operators are keenly aware of such complications.

**Figure 3:**
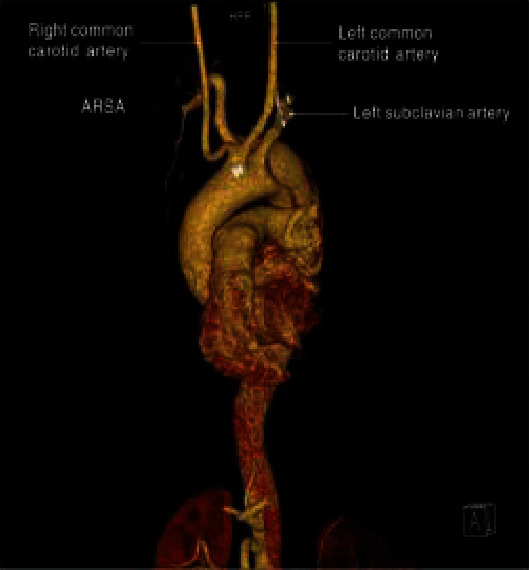
Adapted from “Repair of a type B aortic dissection with a re-vascularization of the aberrant right subclavian artery in an adult patient.”

In addition to appropriate catheter choice, operators must be aware of anatomic variants which can predispose patients to vascular complications. Aberrant right subclavian artery (ARSA) is one such variant (Figure 3), and it can be encountered during a radial catheterization. Though rare, as it is only reported in 0.4-1.8% of the general population; this artery can arise directly from the aortic arch distal to the left subclavian artery [5], instead from the brachiocephalic artery. ARSA does pose an increased risk for vessel trauma especially since its presence is often unbeknownst to the operator.

One of the most feared consequences of vessel trauma is mediastinal hematoma, and most patients present with chest pain, bleeding, or hemodynamic instability immediately after cardiac catheterization. Odynophagia, however, as a chief complaint is extremely rare. In our case, a likely injury of an ARSA led to a mediastinal hematoma which was only discovered when the patient complained of odynophagia in recovery. This complication was not detected on initial electrocardiography or echocardiography. Despite this, the physicians involved in the procedure maintained a high index of suspicion for possible hematoma which was discovered on CT scan. The anatomic variant ARSA which was only discovered postprocedure likely placed this patient at an increased risk for this complication.

Although the patient was able to be managed with supportive care in this scenario, this may not always be possible. Prompt recognition and treatment are imperative in treating this complication. The aforementioned case highlights the fact that unusual complaints such as swallowing dysfunction following cardiac catheterization should not be ignored but rather alert operators to evaluate for potential rare complications such as mediastinal hematoma.

## 4. Conclusion

Mediastinal hematoma is a known but rare complication of transradial cardiac catheterization and is related to vessel trauma during instrumentation [3]. We presented a case where the routine use of 5 French and 6 French catheters led to an ARSA injury and subsequently a mediastinal hematoma. This raises the question of whether a transfemoral approach is better suited for these patients if this aberrancy is identified prior to the procedure.

This patient, however, had a previously unidentified vascular anomaly (ARSA), which likely predisposed her to this complication despite a seemingly uncomplicated transradial catheterization. Prompt recognition of unusual symptoms postprocedure, appropriate hemodynamic and airway management, and possible endovascular repair of the injured vessel are all components of successful management of this potentially life-threatening condition. In addition, a post catheterization angiography of the aberrancy may be helpful to assess for any injury to the vessel prior to sending the patient to recovery if it is recognized during the procedure.

In this case, ARSA was not identified during the catheterization and there was no reported difficulty in engaging the coronaries which would have otherwise alerted the operator. Because of this, operators must have the knowledge of this anatomic aberrancy even if undetected during the procedure. In addition, when vascular injury is suspected, there are a wide variety of symptoms which should raise concern postprocedure, and odynophagia is one such presentation which should not be ignored. This patient's complaint of odynophagia was the direct result of a mediastinal hematoma, and this association has not been previously reported in the literature to the best of our knowledge.
